# The Genus *Nerine* Herb. (Amaryllidaceae): Ethnobotany, Phytochemistry, and Biological Activity

**DOI:** 10.3390/molecules24234238

**Published:** 2019-11-21

**Authors:** Lucie Cahlíková, Nina Vaněčková, Marcela Šafratová, Kateřina Breiterová, Gerald Blunden, Daniela Hulcová, Lubomír Opletal

**Affiliations:** 1 ADINACO Research Group, Department of Pharmaceutical Botany, Faculty of Pharmacy, Charles University, Heyrovského 1203, 500 05 Hradec Králové, Czech Republic; benen7aa@faf.cuni.cz (N.V.); breiterk@faf.cuni.cz (K.B.); hulcovd@faf.cuni.cz (D.H.); opletal@faf.cuni.cz (L.O.); 2 Department of Pharmacognosy, Faculty of Pharmacy, Charles University, Heyrovského 1203, 500 05 Hradec Králové, Czech Republic; safratom@faf.cuni.cz; 3 School of Pharmacy and Biomedical Sciences, University of Portsmouth, Portsmouth, Hampshire P01 2DT, UK; gblunden10@gmail.com

**Keywords:** Amaryllidaceae, *Nerine*, *Nerine bowdenii*, folk medicine, Alzheimer’s disease, antitumor activity

## Abstract

*Nerine* Herbert, family Amaryllidaceae, is a genus of about 30 species that are native to South Africa, Botswana, Lesotho, Namibia, and Swatini (formerly known as Swaziland). Species of *Nerine* are autumn-flowering, perennial, bulbous plants, which inhabit areas with summer rainfall and cool, dry winters. Most *Nerine* species have been cultivated for their elegant flowers, presenting a source of innumerable horticultural hybrids. For many years, species of *Nerine* have been subjected to extensive phytochemical and pharmacological investigations, which resulted in either the isolation or identification of more than fifty Amaryllidaceae alkaloids belonging to different structural types. Amaryllidaceae alkaloids are frequently studied for their interesting biological properties, including antiviral, antibacterial, antitumor, antifungal, antimalarial, analgesic, cytotoxic, and cholinesterase inhibition activities. The present review aims to summarize comprehensively the research that has been reported on the phytochemistry and pharmacology of the genus *Nerine*.

## 1. Introduction

The monocotyledonous family Amaryllidaceae consists of ca. 85 genera and 1100 species, and is one of the 20 most important alkaloid-containing plant families. Amaryllidaceae species are widely distributed over the tropical and warm regions of the world, and have not only been cultivated as ornamental plants for their colorful flowers and fragrant oils, but also for their extensive use as folk medicines against various diseases in many countries and areas [1]. The medical properties of these plants were already known in the fourth century B.C., when Hippocrates of Cos used oil from the daffodil *Narcissus poeticus* L. for the treatment of uterine tumors [2]. Since the isolation of the first alkaloid, lycorine, from *Narcissus pseudonarcissus* in 1877, up to now, more than 600 structurally diverse alkaloids have been isolated from plants of this family with a wide range of interesting biological activities, including antitumor, antibacterial, antifungal, antimalarial, antiviral, analgesic, and acetylcholinesterase inhibitory activities [3,4,5].

The present review summarizes phytochemical studies carried out on the genus *Nerine*, focusing on the occurrence, isolation, identification, and biological activities of secondary metabolites.

## 2. The Genus *Nerine*: Occurrence and Ethnobotany

The genus *Nerine* Herb. is a member of the class Monocotyledonae, order Asparagales, family Amaryllidaceae, and tribe Amaryllideae [6,7]. *Nerine* species form a group of bulbous perennial plants closely related to the genera *Brunsvigia* Heist. and *Hessea* Herb [8]. The first mention of *Nerine* plants was in 1635 when J. Cornut described *Nerine sarniensis* as *Narcissus japonicus rutilo flore*. In 1753 Linnaeus included this species under the name *Amaryllis sarniensis*, and in 1820 the genus *Nerine* was established by Herbert [8]. 

*Nerine*, one of the largest genera within the Amaryllidaceae family, comprises about 30 autumn-flowering perennial bulbous species, confined to temperate regions of Southern Africa, Botswana, Lesotho, Namibia, and Swatini (formerly known as Swaziland), with summer rainfall and cool, dry winters [9]. The exploitation of plants from this genus in the traditional medicinal practices of the indigenous people of southern Africa has been documented [10]. In particular, the southern Sotho and Zulu tribes have used decoctions of the bulbs in the treatment of coughs and colds, renal, and hepatic conditions, to relieve back pain and as a remedy for infertility [11]. Most *Nerine* species have been cultivated for their elegant flowers, presenting a source of innumerable horticultural hybrids [8]. 

## 3. Phytochemistry

**Figure 1:**
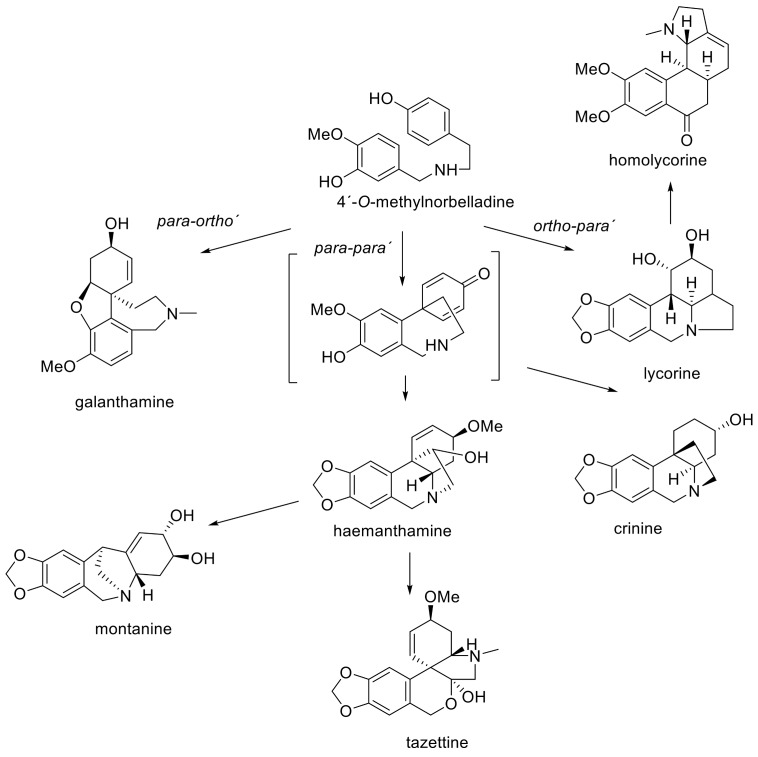
Biosynthetic pathway of main structural types of Amaryllidaceae alkaloids identified in the genus *Nerine* [3,13].

From approximately 30 *Nerine* species described, only eleven have been chemically investigated (Table 1). Particular attention has been given to the study of alkaloids, and only little to other constituents. The Amaryllidaceae alkaloids (AA) are largely restricted to the family Amaryllidaceae, specifically the subfamily Amaryllidoideae [12]. Some noteworthy exceptions are the collection of alkaloids that have been found in the genus *Hosta*, which is in the order Asparagales, along with Amaryllidaceae [12]. Amaryllidaceae alkaloids are derived from the aromatic acids phenyalanine and tyrosine, which are used to produce key intermediates in the biosynthesis of the AA 4′-*O*-methylnorbelladine [13]. According to the name of this key intermediate, this biosynthetic pathway of AA is called the norbelladine pathway. 4′-*O*-Methylnorbelladine undergoes three types of intramolecular oxidative couplings: *ortho-para′, para-para′,* and *para-ortho′*, which leads to the formation of the basic structural types of AA (Figure 1). The *ortho-para′* phenolic coupling gives rise to the lycorine and homolycorine types of AA via norpluviine. A reoxidation of the carbon atom in the central nitrogen containing ring leads to ring opening, following an intramolecular rotation and hemiacetal formation, to form homolycorine type alkaloids. A similar oxidation, as described previously, starts from haemanthamine, which is a direct product of a *para-para′* phenolic coupling. The oxidation results in an epimeric mixture of haemanthidine and epihaemanthidine. Tazettine is then formed by irreversible intramolecular rotation via pretazettine, a direct biosynthetic precursor of tazettine. *Para-ortho′* coupling leads to the formation of the pharmaceutically important alkaloid galanthamine. It is proposed that the *para-ortho′* oxidative phenol coupling of the key intermediate, followed by a spontaneous closure of an ether bridge results in *N*-demethylnarwedine, which is subsequently reduced to norgalanthamine, and finally *N*-methylated to form galanthamine [14]. The belladine-type alkaloids are thought to originate by the simple methylation of norbelladine. A simplified norbelladine pathway of the main structural types of AA identified in the genus *Nerine* is summarized in Figure 1.

The processes used for the extraction and purification of AA from the genus *Nerine* usually include either extraction by boiling under reflux or maceration at room temperature of either minced fresh bulbs or other dried plant parts with 95% ethanol or methanol. The ethanol/methanol extract is then filtered, concentrated, acidified to pH 1.5–2.0, and subjected to a cleaning process including extraction with different organic solvents (e.g., diethyl ether, chloroform, dichloromethane, ethylacetate), changes of pH to 9–10 by the addition of a solution of either ammonia, or 10% Na_2_CO_3_, which results in the preparation of a concentrated alkaloidal extract. This extract is then fractionated on a silica gel column, an alumina column, by HPLC, and preparative TLC to obtain AA in pure form [11,15,16,17,18,19,20,21]. 

**Figure 2:**
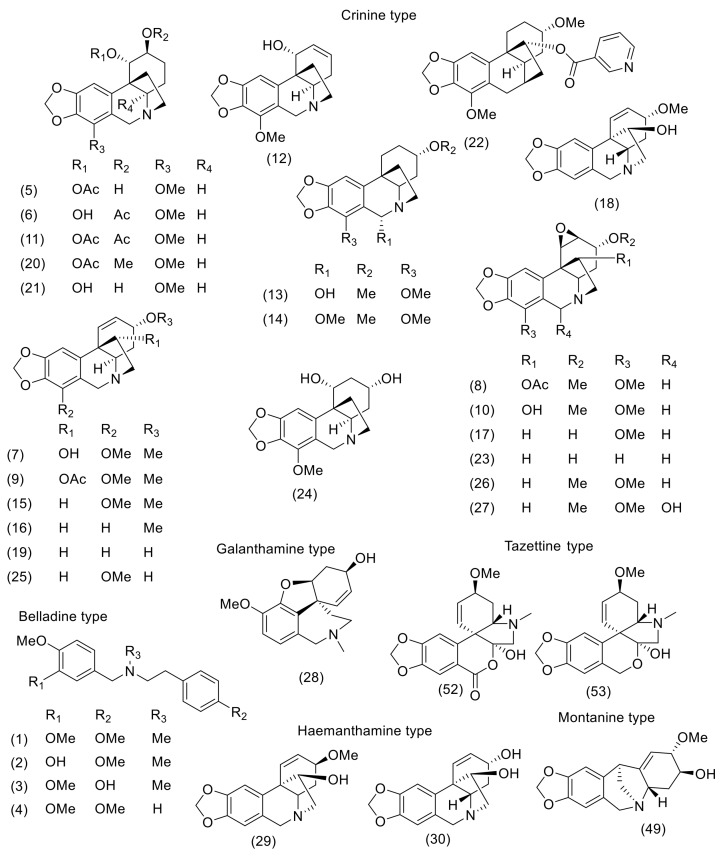
Amaryllidaceae alkaloids of crinine-, galanthamine-, belladine-, tazettine-, haemanthamine, and montanine-type reported in *Nerine* species.

**Figure 3:**
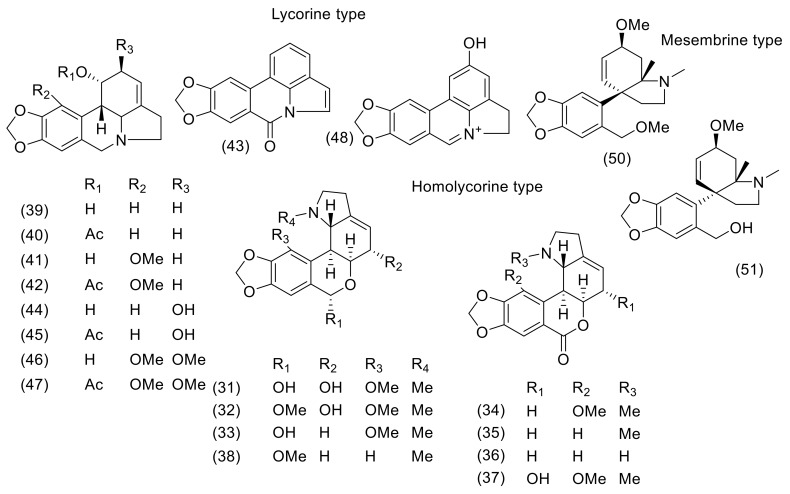
Amaryllidaceae alkaloids of lycorine-, homolycorine-, and mesembrine type reported in *Nerine* species.

Altogether, 53 Amaryllidaceae alkaloids of various structural types have been either isolated or identified in the studied *Nerine* plants (Table 1). The described alkaloids belong to belladine, crinine, lycorine, haemanthamine, homolycorine, mesembrine, montanine, and tazettine structural types (Figure 2, Figure 3, Table 1). The most studied plant is *N. bowdenii* W. Watson, an endemic Amaryllidaceae species native to KwaZulu-Natal, Drakensberg, and Eastern Cape Provinces in South Africa [15]. Different phytochemical studies led to the isolation of about 30 Amaryllidaceae alkaloids in bulbs of *N. bowdenii* (Table 1). Most of the isolated alkaloids belong to the crinine, lycorine, and belladine structural types. Based on GC/MS analysis reported in 2011 [16], the alkaloid pattern of bulbs of *N. bowdenii* was dominated by belladine (**1**), ambelline (**7**), and *N*-demethylbelladine (**4**) [16]. A detailed phytochemical study in 2015 led to the isolation of twenty-two AA from fresh bulbs of *N. bowdenii*. The isolated compounds have been studied for their biological activity connected with the potential treatment of Alzheimer′s and oncological diseases [17].

*N. filifolia* Baker is an evergreen plant, with small bulbs and thin, thread-like leaves. It flowers between September and November and occurs in the Eastern Cape, Transkei, Orange Free State, Swatini, and Mpumalanga. *N. filifolia* was examined for its alkaloidal constituents [16], and, following the usual extractive and chromatographic procedures, eight alkaloids, as well as phenol, were isolated. The known compounds belladine (**1**), 11-*O*-acetylambelline (**9**), and undulatine (**26**) were isolated as the main compounds, in addition to the minor components: Ambelline (**7**) and 6α-hydroxybuphandrine (**13**). *N*-Demethylbelladine (**4**), 6α-methoxybuphandrine (**14**) and filifoline (**22**, the 11-*O*-nicotinyl analogue of ambelline) were reported for the first time as natural products. A small quantity of galanthamine (**28**) was detected in one phytochemical analysis of *N. filifolia* [22]. 

In another study, GS/MS was employed for the determination of compounds present in the concentrated alkaloidal extract of *N. filifolia*, prepared by using acidic-basic conditions of alkaloids and final extraction with ethylacetate. Seven compounds showed MS fragmentation patterns characteristic of Amaryllidaceae alkaloids; six of them were identified as masonine (**35**), *N*-demethylmasonine (**36**), caranine (**39**), lycorine (**44**), crinine (**19**), acetylcaranine (**40**), and *O*-methyloduline (**38**) (Table 1, Figure 3). The alkaloidal pattern was dominated by homolycorine- type alkaloids [25].

*N. huttoniae* Schönland is a summer growing, evergreen species with large bulbs and sharp-shaped, shiny, dark green leaves. It occurs in the western part of Eastern Cape Province of South Africa where it grows in colonies either near riverbanks or in seasonally damp depressions [46]. In the screening for CNS-active alkaloids from the family Amaryllidaceae, the alkaloidal extract from *N. huttoniae* showed inhibitory activity against acetylcholinesterase. Four alkaloids were isolated from this plant, lycorine (**44**), tazettine (**53**), oxokrigenamine (**34**), and 6-*O*-methylkrigeine (**32**), of which 6-*O*-methylkrigeine (**32**) was identified in an Amaryllidaceae plant for the first time [18].

The alkaloid pattern of *N. filamentosa* W.F. Barker, studied by a GC/MS technique, was dominated by three crinine-type alkaloids: Undulatine (**26**), buphanamine (**12**), and ambelline (**7**), and one lycorine-type alkaloid, tentatively identified as acetylparkamine (**47**) (Table 1, Figure 2, Figure 3) [25]. Both parkamine (**46**) and acetylparkamine (**47**) were identified by comparing their MS with those of already known compounds of the same structural type caranine (**39**), acetylcaranine (**40**), falcatine (**41**), and acetylfalcatine (**42**), which were available in the NIST library [25].

Phytochemical investigations of *N. undulata* (L.) Herb led to the isolation and identification of three AA, lycorine (**44**), ambelline (**7**), and undulatine (**26**) [26]. The alkaloid pattern of *N. undulata*, studied by GC/MS, was dominated by two crinine-type alkaloids—undulatine (**26**), and buphanamine (**12**), and two unidentified alkaloids [27]. Some other minor alkaloids were also identified—buphanidrine (**15**), ambelline (**7**), bowdesine (**11**), crinamidine (**17**), and flexinine (**23**) (Table 1; Figure 2).

*N. sarniensis* (L.) Herb. is considered as one of the most beautiful of all Nerines. It is also known as Guernsey Lily, and is restricted to the Western Cape of South Africa [47]. The alkaloids of this species remain largely unexplored, but, so far, tazettine (**53**), nerinine (**37**), lycorine (**44**), 3-epimacronine (**52**), and sarniensine (**50**) have been isolated [19,20].

An overview of the EI/MS and fragmentation patterns of AA identified in the genus *Nerine* is summarized in Table S1 (Supplementary Material).

## 4. Biological Activity

As mentioned before, AA have shown a wide range of bioactivity, including antiviral, antibacterial, antitumor, antifungal, antimalarial, analgesic, cytotoxic, and insecticidal properties, as well as activity on the central nervous system (CNS), such as hallucinogenic effects, mental disorders, and age-related dementia [48,49].

### 4.1. Biological Activity Connected with Potential Treatment of Alzheimer′s Disease

Nowadays, the ability to inhibit acetylcholinesterase (AChE, E.C. 3.1.1.7) is the most significant activity of AA. This inhibition is mainly used in the treatment of Alzheimer′s disease (AD), which is a major neurodegenerative disease that is clinically manifested by dementia [50]. The cause of this disease is not known, and therefore the pharmacotherapy cannot be as effective as in the case of other positively clarified diseases [51]. From the beginning, AD is characterized by a defect in the central acetylcholinesterase system [52]. The central cholinesterase inhibitors are the most important and most effective drugs. They are used in the therapy of the early and intermediate states of dementia [53].

One of the most important substances with the ability to inhibit AChE is an alkaloid isolated from the family Amaryllidaceae, specifically galanthamine (**28**). It was isolated first from the bulbs of *Galanthus woronowii* Losinsk. and later from other plants of the family [54].

From the launch of galanthamine (**28**) into clinical practice, other Amaryllidaceae alkaloids received attention as potential AChE inhibitors, such as AA from the genus *Nerine*. Different alkaloidal extracts of *Nerine* plants have been tested, and some of them showed interesting acetylcholinesterase or butyrylcholinesterase inhibitory activity [16,17,21,25,27,55]. Cholinesterases inhibition activities of the studied *Nerine* extracts and isolated alkaloids are summarized in Table 2.

Among the plants of the family Amaryllidaceae, AChE inhibitory activity is associated mainly with the presence of galanthamine- and lycorine-type alkaloids, which are known to possess such inhibitory activity [56,57]. Lycorine-type compounds are less active inhibitors than the galanthamine type compounds and their activity is associated with a substitution at positions C-1 and C-2 [58]. Crinine-type alkaloids are reported to possess only weak inhibition activities. The haemanthamine- and crinine-type alkaloids differ only in the position of the 5,10b-ethano bridge, but it appears that the configuration of this bridge has no effect on the AChE inhibition activity [59]. Contrary to these conclusions, AChE/BuChE inhibition activity of some crinine-type AA isolated in the last decade is also interesting [15,17,60,61]. Two crinine-type alkaloids, undulatine (**26**) and powelline (**25**), were the most active compounds in the AChE assay of the alkaloids isolated from *N. bowdenii*, with IC_50_ values of 23.5 ± 1.2 µM and 29.1 ± 1.6 µM, respectively [58]. Moreover, undulatine (**26**) has been studied for its mechanism of AChE inhibition, and its penetration through the blood-brain barrier (BBB). Undulatine acts via a mixed inhibition mechanism, and on the basis of the PAMPA-BBB method, is able to cross the BBB by passive permeation [60]. Another interesting AChE inhibitor within the AA lycorine-type is ungeremine (**48**; IC_50_ = 0.35 µM), isolated by preparative HPLC coupled on-line to a flow assay system. This alkaloid demonstrated stronger activity than galanthamine (IC_50_ = 2.2 µM) [21]. Further AA isolated from *Nerine* species have been recognized as inactive.

Targeting the cytosolic serine peptidase, prolyl oligopeptidase (POP; E.C. 3.4.21.26) is currently discussed as an emerging therapy of AD [62]. POP cleaves peptide bonds at the carboxyl end of proline. It is widely distributed in the organs of the body, including the brain [63]. Previous studies indicated that POP activity is involved in key physiological functions, such as learning and memory, cell division and differentiation, and signaling transduction, as well as in some psychiatric disorders [64]. Indeed, some POP inhibitors have been experimentally found to be efficacious anti-dementia drugs [62]. AA isolated from *N. bowdenii* have been studied for POP inhibition activity, and the most interesting inhibition has been demonstrated by 4′-*O*-demethylbelladine (**2**), buphanidrine (**15**), and 1-*O*-acetyllycorine (**45**), with IC_50_ values of 370 ± 30, 370 ± 40, and 450 ± 50 µM, respectively [17]. Some Amaryllidaceae alkaloids have been previously tested for their POP inhibition activity; the best results have been shown by the lycorine type alkaloid 9-*O*-demethylgalanthine (IC_50_ = 150 ± 20 µM), isolated from *Zephyranthes robusta* [65]. The lycorine structure seems to be interesting for POP inhibition, but a wider range of compounds of either natural origin or semisynthetic analogues must be tested first.

### 4.2. Antitumor Activity

Oncological diseases are one of the leading causes of death in the developed countries. Among numerous species that have been investigated in the search for small molecule constituents with potential in therapy, plants of the Amaryllidaceae family have been particularly fruitful [66]. Although antitumor activities are detectable across a series of alkaloids of the Amaryllidaceae, the most promising are members of the lycorine-, haemanthamine-, and pancratistatine-type [41,67,68]. The interest in lycorine and the Amaryllidaceae alkaloids was more recently renewed thanks to the discovery of their strong anticancer activity. Lycorine (**44**) appeared to be the strongest anticancer compound in a single-digit micromolar range and showed a cytostatic rather than cytotoxic effect. Lycorine displays significant in vitro cytotoxic activity against several different types of cancer cell lines, including Rauscher, HeLa, P-388, U-373, HepG2, 3T3, KB, Molt-4, HT-1080, COL-2, and ZR-75-1 [69]. Furthermore, lycorine (**44**) displayed significant therapeutic benefit at nontoxic doses in mice bearing grafts of the B16F10 melanoma model. A recent in vitro study demonstrated that lycorine inhibits the proliferation, migration, invasion, and survival of various prostate cancer cell lines, such as PC-3M, DU145, LNCaP, and 22RV1, with IC_50_ values ranging from 5 to 10 µM [70]. Additional in vivo experiments showed that the intraperitoneal administration (i.p.) of lycorine (**44**) (5 or 10 mg/kg/day) reduces both the weight and volume of ectopically implanted PC-3M subcutaneous xenografts by about 80% and exhibits no obvious systematic toxicity. Lycorine appeared to be an excellent lead for the generation of compounds which are able to combat cancers naturally resistant to the proapoptotic stimuli, such as glioblastoma, melanoma, nonsmall-cell lung cancers, and metastatic cancers [51]. Lycorine′s mechanism of action has been studied in detail on leukemia (human cell line HL-60), human multiple myeloma cell line KM3, and myelogenous leukemia K562 [71]. Alkaloids isolated from *N. bowdenii* were screened for their cytotoxic activity against p53-mutated Caco-2 and HT-29 colorectal adenocarcinoma cells. At the same time, healthy small intestine cells (FHs 74 Int) were used to determine overall toxicity against noncancerous cells [17,68]. Among the tested compounds, haemanthamine (**29**) and buphanisine (**16**) demonstrated the most potent cytotoxic effect against both tested cell lines, while showing significantly lower toxicity against normal cells (Table 3). Other evaluated alkaloids were determined as non-cytotoxic (IC_50_ > 10 µM). Haemanthamine, one of the most abundant AA within the family Amaryllidaceae, has attracted serious researcher attention, and its anticancer potential has been proved with different types of human cancer cell, such as T-lymphoblast leukemia, promyelocytic leukemia, hepatocellular carcinoma, lung carcinoma, esophageal squamous carcinoma, brain glioma, melanoma, and cervical adenocarcinoma [58,72,73,74,75,76,77]. Interesting is its ability to overcome cancer cell resistance, and to initiate apoptotic cell death [67]. Recently, the effect of haemanthamine (**29**) on cell cycle progression and apoptosis in p53-negative human leukemic Jurkat cells has been studied [77]. The obtained results indicated that haemanthamine treatment decreases cell viability and mitochondrial membrane potential, leads to a decline in the percentage of cells in the S phase of the cell cycle, induces apoptosis detected by Annexin V staining, and increases caspase activity. Dose dependent apoptosis was cross verified by fluorescence and bright field microscopy through Annexin V/propidium iodine staining and morphological changes that characteristically attend programmed cell death. Treatment with haemanthamine (**29**) led to accumulation of cells preferentially at the G1 and G2 stages of the cell cycle with increased p16 expression and Chk1 Ser345 phosphorylation [77]. The in vivo anticancer potential of haemanthamine (**29**) has been preliminary quantified by a study performed using an Ehrlich tumor-bearing mice model. In order to characterize the in vivo effect, survival of recipient mice was monitored. The mean overall survival time without therapy was 15.4 days [76]. Regrettably, the Ehrlich tumor showed unrestricted progression after haemanthamine treatment at concentrations of 10, 20, or 30 mg/kg. Moreover, Ehrlich tumor-bearing mice that had received haemanthamine (**29**) had no significant survival advantage compared with the negative control groups [76]. These in vivo findings were inconsistent with previous experiments by Furusava et al. [78]. The study reported a prolonged survival of mice with established Rauscher leukemia after treatment with haemanthamine (**29**) [75].

From the perspectives for development of new anticancer drugs, haemanthamine has a serious advantage in comparison with other promising cytotoxic AA, such as narciclassine and pancratistatine, as its solubility is higher than 1 mg/mL and it contains a basic nitrogen atom, allowing its potential administration in salt form [78].

Filifoline (**22**) isolated from *N. filifolia* displayed no cytotoxicity to myoblast (L6) cells [11].

### 4.3. Further Biological Activity

In a preliminary study, the organic extract of *N. sarniensis* bulbs showed strong adulticidal and larvicidal activity against *Aedes aegypti*, which is the major vector of the arboviruses responsible for dengue and yellow fevers and Zika viral diseases, which are major threats to public health. There is no approved vaccine against Zika virus [79], and the vaccine against dengue has only been registered recently [80]. Subsequently, the isolated alkaloids from *N. sarniensis* were assayed against *A. aegypti*. None showed mortality against the 1st instar larvae at the tested concentrations. In the adult topical bioassays, only crinsarnine (**20**) displayed strong adulticidal activity with an LD_50_ = 2.29 ± 0.049 μg/mosquitos [19]. As for the structure–activity relationship, the pretazettine scaffold in sarniensinol (**51**) and sarniensine (**50**) and the crinine one in crinsarnine (**20**) and bowdesine (**11**) proved to be important for their activity in the same way as the pyrolle[*de*]phenanthridine scaffold was for the activity of hippadine (**43**) and 1-*O*-acetyllycorine (**45**) [19,20]. Among the pretazettine group, either the opening of the B ring or the presence of a B ring lactone, as well as the *trans*-stereochemistry of the A/B ring junction appear to be important for the activity. In the crinine type of alkaloids, the substituent at C-2 seems to play a role in their activity [19,20].

## 5. Conclusions

This review summarizes ethnobotanical, phytochemical, and pharmacological information about the genus *Nerine*, family Amaryllidaceae, and closely related to the genera *Brunsvigia* Heist. and *Hessea* Herb. From approximately 30 *Nerine* species described, only eleven have been chemically investigated. Particular attention has been given to the study of alkaloids, and only little to other constituents. Altogether 53 Amaryllidaceae alkaloids of various structural types have been either isolated or identified in the studied *Nerine* plants, so far. Some of the isolated alkaloids have been subjected to several biological assays connected with the potential treatment of neurodegenerative and oncological diseases. The most promising alkaloids isolated from *Nerine* species are haemanthamine and lycorine, which are being intensively studied for their antitumor properties. Adulticidal and larvicidal activity against *Aedes aegypti* of some alkaloids has also been studied. In the light of the presented overview of scientific data, the genus *Nerine* can be recognized as an interesting source of different structural types of Amaryllidaceae alkaloids with a wide range of biological activities.

## Figures and Tables

**Table 1:** Alkaloids reported in the genus *Nerine.*

	*N. Bowdenii*	*N. Corusca*	*N. Falcata*	*N. Filamentosa*	*N. Filifolia*	*N. Flexuosa*	*N. Huttoniae*	*N. Krigeii*	*N. Laticoma*	*N. Sarniensis*	*N. Undulata*	Ref for NMR, MS Data
***Belladine-type***												
Belladine (**1**)	[16,17,23]				[11]							[11,16]
4′-*O*-Demethylbelladine (**2**)	[17]											[17]
6-*O*-Demethylbelladine (**3**)	[17]											[17]
*N*-Demethylbelladine (**4**)	[16]				[11]							[11,16]
***Crinine-type***												
1-*O*-Acetylbulbisine (**5**)	[17]											[24]
Acetylnerbowdine (**6**)	[16]											[16]
Ambelline (**7**)	[15,16,17,23]			[25]	[11]						[26,27]	[25,28]
11-*O*-Acetyl-1,2-epoxy- ambelline (**8**)	[16]											[16,29]
11-*O*-Acetylambelline (**9**)	[15,16,17]				[11]							[16,30]
1,2-Epoxyambelline (**10**)	[17]											[17,29]
Bowdensine (**11**)	[16,23]									[19]	[27]	[16,19]
Buphanamine (**12**)	[16,17]			[25]							[27]	[16,31]
6α-Hydroxy-buphanidrine (**13**)					[11]							[11]
6α-Methoxy-buphanidrine (**14**)					[11]							[11]
Buphanidrine (**15**)	[16,17,23]										[27]	[16,30]
Buphanisine (**16**)	[15,16,17,23]											[16,30]
Crinamidine (**17**)	[15,16,23,32]	[33]				[32]					[27]	[16,33]
Crinamine (**18**)	[16,23]											[16,33]
Crinine (**19**)	[15,16,23]				[25]							[16,30,33]
Crinsarnine (**20**)										[19]		[19]
Deacetylbowdesine (**21**)	[17]											[17]
Filifoline (**22**)	[15]				[11]							[11]
Flexinine (**23**)											[27]	[27,29]
Nerbowdine (**24**)	[23]											[23]
Powelline (**25**)	[15,16]											[16,33]
Undulatine (**26**)	[15,16,17,23]			[25]	[11]						[26,27]	[16,28,29,30]
6-Hydroxyundulatine (**27**)	[17]											[29]
***Galanthamine-type***												
Galanthamine (**28**)					[22]							[25,34]
***Haemanthamine-type***												
Haemanthamine (**29**)	[17]										[27]	[25,35]
Hammayne (**30**)	[17]											[33]
***Homolycorine-type***												
Krigeine (**31**)								[36]				[36]
6-*O*-Methylkrigeine (**32**)							[18]					[18]
Krigenamine (**33**)								[36]				[36]
Oxokrigenamine (**34**)							[18]					[18]
Masonine (**35**)					[25]							[25,37]
*N*-Demethylmasonine (**36**)					[25]							[25,37]
Nerinine (**37**)								[36]				[38,39]
*O*-Methyloduline (**38**)					[25]							[25,37]
***Lycorine-type***												
Caranine (**39**)	[15,16]		[40]		[25]				[40]			[16,41]
Acetylcaranine (**40**)	[15,16]				[25]							[16,28]
Falcatine (**41**)			[40]						[27]			[27,40]
Acetylfalcatine (**42**)	[16]											[16]
Hippadine (**43**)										[19]		[42,43]
Lycorine (**44**)	[23]		[40]	[25]	[25]		[18]	[36]	[40]		[26]	[18,25,41]
1-*O*-Acetyllycorine (**45**)	[15,23]									[19]		[41,44]
Parkamine (**46**)				[25]								[25]
Acetylparkamine (**47**)				[25]								[25]
Ungeremine (**48**)	[21]											[21,41]
***Montanine-type***												
Montanine (**49**)											[27]	[27,31]
***Mesembrine-type***												
Sarniensine (**50**)										[20]		[20]
Sarniensinol (**51**)										[19]		[19]
***Tazettine-type***												
3-Epimacronine (**52**)										[19]	[27]	[27,45]
Tazettine (**53**)	[15]						[18]			[19]	[27]	[18,31]

**Table 2:** AChE, BuChE, and POP inhibitory activity of the alkaloidal extracts of *Nerine* species and isolated alkaloids.

Plant Species	AChE, IC_50_ (µg/mL)	BuChE, IC_50_ (µg/mL)	POP, IC_50_ (µg/mL)	Reference
*N. bowdenii*	87.9 ± 3.571% inhibition of AChE	14.8 ± 1.1	n.t.	[27,55]
*N. filamentosa*	21.6 ± 1.1	13.0 ± 0.7	n.t.	[25]
*N. filifolia*	18.5 ± 0.5	58.6 ± 1.3	n.t.	[25]
*N. undulata*	14.3 ± 1.2	33.9 ± 1.9	n.t.	[16]
	**AChE, IC_50_ (µM)**	**BuChE, IC_50_ (µM)**	**POP, IC_50_ (µM)**	
Undulatine (**26**)	23.5 ± 1.2	>1000	>1000	[17]
Poweline (**25**)	29.1 ± 1.6	394.2 ± 4.8	770 ± 20	[17]
Ungeremine (**48**)	0.35	n.t	n.t	[21]
6-*O*-Demethylbelladine (**3**)	223.2 ± 23.6	115.7 ± 10.1	660 ± 90	[17]
4′-*O*-Demethylbelladine (**2**)	606.8 ± 74.2	30.7 ± 4.0	370 ± 30	[17]
1-*O*-Acetyllycorine (**45**)	> 1000	176.2 ± 14.2	450 ± 50	[17]
Crinamidine (**17**)	230.1 ± 9.8	> 1000	790 ± 60	[17]
Galanthamine*	1.7 ± 0.1	42.3 ± 1.3	> 1000	[17]
Berberine*	n.t.	n.t.	140 ± 2	[17]

*standard.

**Table 3:** Cytotoxicity of AA isolated from *N. bowdenii* against two cancer cell lines and one noncancerous gastrointestinal cell line [17,68].

	Cancer Cells		Normal Cells
Compound	Caco-2 IC_50_ (µM)	HT-29 IC_50_ (µM)	FHs 74 Int IC_50_ (µM)
Ambelline (**7**)	74.1 ± 1.1	50.2 ± 1.2	89.8 ± 6.5
11-*O*-Acetylambelline (**9**)	> 100	> 100	> 100
1-*O*-Acetylbulbisine (**5**)	33.4 ± 2.9	47.9 ± 1.6	61.3 ± 8.8
Acetylcaranine (**40**)	29.5 ± 0.6	19.2 ± 1.2	66.1 ± 6.8
Buphanamine (**12**)	53.5 ± 0.7	47.6 ± 2.2	> 100
Buphanisine (**16**)	8.6 ± 0.2	5.3 ± 1.7	22.8 ± 2.6
Caranine (**39**)	64.4 ± 4.5	46.6 ± 1.9	> 100
Crinine (**19**)	64.5± 17.8	50.8 ± 1.4	> 100
Haemanthamine (**29**)	1.0 ± 0.1	0.6 ± 0.0	19.5 ± 8.9
Hamayne (**30**)	17.2 ± 0.9	12.4 ± 0.3	53.3 ± 7.4
Tazettine (**53**)	22.8 ± 3.3	23.4 ± 2.0	71.1 ± 5.2
Undulatine (**26**)	51.7 ± 1.1	53.4 ± 2.2	70.4 ± 6.8

## Supplementary Materials

The following are available online, Supplementary data: EI/MS fragmentation patterns.
