# Supplementary material for: The Genus Nerine Herb. (Amaryllidaceae): Ethnobotany, Phytochemistry, and Biological Activity
Source: Molecules. 2019 Nov 21;24(23):4238. doi: 10.3390/molecules24234238 (PMC6930486; doi:10.3390/molecules24234238)
Supplement: Supplementary file 1 [file molecules-24-04238-s001.pdf]

Supplementary Material for:

The genus *Nerine* Herb. (Amaryllidaceae): Ethnobotany, phytochemistry and biological activity

Lucie Cahlíková[1*], Nina Vaněčková[1], Marcela Šafratová[2], Kateřina Breiterová[1], Gerald Blunden[3], Daniela Hulcová[1], Lubomír Opletal[1]

[1] ADINACO Research Group, Department of Pharmaceutical Botany, Faculty of Pharmacy, Charles University, Heyrovského 1203, 500 05 Hradec Králové, Czech Republic

[2] Department of Pharmacognosy, Faculty of Pharmacy, Charles University, Heyrovského 1203, 500 05 Hradec Králové, Czech Republic

[3] School of Pharmacy and Biomedical Sciences, University of Portsmouth, Portsmouth, Hampshire, UK

* Correspondence: cahlikova@faf.cuni.cz; Tel.: +420-495067311; Fax: +420-495067162

	[M$^+$] and characteristic ions, m/z (% relative intensity)	Ref for MS data
Belladine-type		
Belladine (**1**)	315 (2), 194 (70), 152 (90), 151 (100), 135 (10), 121 (18), 107 (18)	[1]
4´-*O*-Demethylbelladine (**2**)	301 (1), 181 (70), 137 (100), 121 (10), 107 (15), 93 (15)	[2]
6-*O*-Demethylbelladine (**3**)	301 (1), 194 (68), 151 (100), 135 (12), 121 (15), 107 (17)	[2]
N-Demethylbelladine (**4**)	301 (2), 299 (3), 273 (1), 195 (6), 180 (20), 151 (100), 138 (4), 128 (2), 121 (9), 107 (6)	[1,3]
Crinine-type		
1-*O*-Acetylbulbisine (**5**)	EIMS not available in literature; HRESIMS (TFA salt): 362.1581 [M + H]$^+$	[4]
Acetylnerbowdine (**6**)	361 (75), 318 (30), 302 (60), 273 (20), 254 (50), 244 (25), 43 (100)	[1]
Ambelline (**7**)	331(100), 316(5), 299(30), 287(55), 270(25), 260(50), 257(35), 255(23),241(27)	[5]
11-*O*-Acetyl-1,2-epoxyambelline (**8**)	389 (60), 330 (75), 316 (100), 274 (30), 256 (55), 231 (40), 228 (12), 205 (25), 203 (27), 190 (18)	[1,6]
11-*O*-Acetylambelline (**9**)	373 (100), 358 (15), 342 (15), 330 (10), 314 (70), 313 (69), 298 (30), 282 (55),270 (25)	[1]
1,2-Epoxyambelline (**10**)	347 (33), 318 (100), 274 (25), 244 (24), 231 (27), 205 (47), 189 (14), 173 (11), 115 (18)	[6]
Bowdensine (**11**)	403 (82), 344 (80), 288 (75), 284 (50), 272 (40), 230 (40), 217 (42), 189 (28), 173 (50), 43 (100)	[1]
Buphanamine (**12**)	301 (100), 286 (12), 284 (14), 272 (14), 256 (18), 231 (25), 218 (18), 204 (18)	[1]
6α-Hydroxybuphanidrine (**13**)	331 (54), 299 (22), 287 (49), 276 (100), 261 (36), 255 (37), 243 (20), 217 (34), 203 (20), 115 (38), 77 (36), 56 (75)	[3]
6α-Methoxybuphanidrine (**14**)	345 (65), 330 (32), 315 (27), 314 (100), 290 (33), 287 (41), 259 (88), 227 (30), 129 (24), 97 (26), 81 (44), 69 (97)	[3]
Buphanidrine (**15**)	315 (100), 300 (26), 287 (30), 272 (10), 260 (45), 245 (60), 231 (30), 228 (22)	[1]
Buphanisine (**16**)	285 (100), 270 (23), 253 (25), 230 (28), 215 (80), 201 (22), 187 (20), 157 (15)	[1]
Crinamidine (**17**)	317 (75), 288 (100), 274 (5), 259 (22), 258 (25), 243 (28), 244 (30), 230 (25), 217 (40), 205 (40)	[1]
Crinamine (**18**)	301 (1), 269 (100), 240 (35), 225 (20), 224 (25), 211 (17), 181 (58)	[1]
Crinine (**19**)	271 (100), 254 (8), 242 (8), 228 (25), 216 (14), 199 (48), 187 (43)	[1]
Crinsarnine (**20**)	EIMS not available in literature; HRESIMS: 376.1766 [M + H]$^+$	[7]
Deacetylbowdesine (**21**)	EIMS not available in literature	
Filifoline (**22**)	436(1), 296 (1), 185 (2), 165 (3), 145 (2), 122 (3), 110 (3), 106 (45), 99 (7), 91 (26), 79 (100), 69 (25), 55 (63)	[3]
Flexinine (**23**)	287(55), 258(100), 229(15). 228(20), 215(30), 215(25), 187(45), 186(15), 175(55), 173(30),143(70), 115(85)	[6,8]
Nerbowdine (**24**)	EIMS not available in literature	
Powelline (**25**)	301 (100), 284 (8), 272 (7), 258 (20), 246 (12), 244 (12), 229 (80), 217 (40)	[1]
Undulatine (**26**)	331 (100), 316 (6), 302 (9), 300 (6), 286 (20), 272 (5), 260 (18), 258 (40)	[1]
6-Hydroxyundulatine (**27**)	347 (47), 318 (3), 276 (44), 274 (22), 256 (39), 246 (21), 231 (27), 219 (96), 204 (38), 189 (18), 115 (27, 56 (100)	[6]
Galanthamine-type		
Galanthamine (**28**)	287(90), 286(100), 270(20), 244(30), 230(5), 216(45)	[5]
Haemanthamine-type		
Haemanthamine (**29**)	301(15), 272(100), 240(15), 225(5), 211(15)	[5]
Hammayne (**30**)	287 (5), 259 (18), 258 (100), 214 (10), 186 (14), 181 (14), 115 (13)	[9,10]
Homolycorine-type		
Krigeine (**31**)	EIMS not available in literature	
6-*O*-Methylkrigeine (**32**)	EIMS not available in literature; HRESIMS: 362.1599 [M + H]$^+$	[11]
Krigenamine (**33**)	EIMS not available in literature	
Oxokrigenamine (**34**)	EIMS not available in literature	
Masonine (**35**)	299 (1), 190 (2), 162 (4), 134 (1), 109 (100), 108 (23), 94 (4), 82 (2)	[5]
N-Demethylmasonine (**36**)	285(<1), 162(2), 134(1), 115(1), 95 (100)	[5]
Nerinine (**37**)	347 (-), 330 (7), 329 (3), 236 (1), 221 (9), 191 (2), 109 (100), 94 (2)	[10,12]
O-Methyloduline (**38**)	315 (<1), 284 (2), 250 (1), 175 (10), 109 (100)	[5]
Lycorine-type		
Caranine (**39**)	271 (86), 270 (46), 252 (52), 250 (10), 240 (12), 227 (48), 226 (100), 212 (5)	[1]
Acetylcaranine (**40**)	313 (80), 270 (5), 253 (20), 252 (95), 250 (15), 240 (10), 225 (40), 226 (100)	[1]
Falcatine (**41**)	EIMS not available in literature	
Acetylfalcatine (**42**)	343 (75), 300 (5), 283 (25), 282 (80), 266 (30), 257 (77), 256 (100), 241 (12)	[1]
Hippadine (**43**)	263 (100), 205 (6), 177 (24), 150 (12)	[13]
Lycorine (**44**)	287(35), 286(30), 268(20), 250(15), 227(70), 226(100)	[5,11]
1-*O*-Acetyllycorine (**45**)	329 (52), 268 (37), 250 (24), 240 (9), 226 (100), 192 (3)	[14,15]
Parkamine (**46**)	331 (76), 312 (60), 287 (70), 286 (100), 282 (17), 256 (17), 44 (54)	[5]
Acetylparkamine (**47**)	373 (80), 313 (18), 312 (60), 287 (77), 286 (100), 282 (20), 256 (10)	[5]
Ungeremine (**48**)	EIMS not available in literature; HRAPCI: 266.0817 [M + H]$^+$	[16]
Montanine-type		
Montanine (**49**)	301(100), 270(75), 257(30), 252(20), 223(15)	[8]
Mesembrine-type		
Sarniensine (**50**)	EIMS not available in literature; HRESIMS: 332.1871 [M + H]$^+$	[17]
Sarniensinol (**51**)	EIMS not available in literature; HRESIMS: 318.1712 [M + H]$^+$	[7]
Tazettine-type		
3-Epimacronine (**52**)	329 (30), 314 (25), 245 (100), 225 (15), 201 (80), 139 (10)	[8,10]
Tazettine (**53**)	331(21), 298(20), 247(100), 201(20), 115(23), 70(34)	[11]

References

1. Cahlíková, L.; Zavadil, S.; Macáková, K.; Valterová, I.; Kulhánková, A.; Hošťálková, A.; Kuneš, J.; Opletal, L. Isolation and cholinesterase activity of Amaryllidaceae alkaloids from *Nerine bowdenii*. *Nat. Prod. Commun.* **2011**, *6*, 1827-1830.

2. Vaněčková, N. Study of the inhibitory (toxic) effect of the alkaloids from chosen plants of Amaryllidaceae family on some human enzymatic systems (*in vitro* study) II. Hradec Králové. *Doctoral thesis* **2018**. Faculty of Pharmacy, Charles University.

3. Nair, J.J.; Campbell, W.E.; Brun, R.; Viladomat, F.; Codina, C.; Bastida, J. Alkaloids from *Nerine filifolia*. *Phytochemistry* **2005**, *6*, 373-382.

4. Chen, Ch.K.; Lin, F.H.; Tseng, L.H.; Jiang, Ch.L.; Lee, S.S. Comprehensive study of alkaloids from *Crinum asiaticum* var. *sinicum* assisted by HPLC-DAD-SPE-NMR. *J. Nat. Prod.* **2011**, *74*, 411-419.

5. Cahlíková, L.; Benešová, N.; Macáková, K.; Kučera, R.; Hrstka, V.; Klimeš, J.; Jahodář, L.; Opletal, L. Alkaloids from some Amaryllidaceae species and their cholinesterase activity. *Nat. Prod. Commun.* **2012**, *7*, 571-574.

6. Machocho A., Chhabra S.C., Viladomat F., Codina C., Bastida J.: Alkaloids from *Ammocharis tinneana. Phytochemistry*, **1999**, 51, 1185-1191.

7. Masi, M.; Cala, A.; Tabanca, N.; Cimmino, A.; Green, I.R.; Bloomquist, J.R.; van Otterlo, W.A.L.; Macias, F.A.; Evidente, A. Alkaloids with activity against Zika virus vector *Aedes aegypti* (L.) - Crisarnine and Sarniensinol, two new crinine and mesembrine type alkaloids isolated from the South African plant *Nerine sarniensis*. *Molecules* **2016**, *21*, 1432.

8. Cahlíková, L.; Benešová, N.; Macáková, K.; Urbanová, K.; Opletal, L. GC/MS analysis of three Amaryllidaceae species and their cholinesterase activity, *Nat. Prod. Commun.* **2011**, *6*, 1255-1258.

9. Kobayashi, S.; Tokumoto, T.; Kihara, M.; Imakura, Y.; Shingu, T.; Taira, Z. Alkaloidal constituents of *Crinum latifolium* and *Crinum bulbispermum* (Amaryllidaceae). *Chem. Pharm. Bull.* **1984**, *32*, 3015-3022.

10. de Andrade, J.P.; Giordani, R.B.; Torras-Claveria, L.; Pigni, N.B.; Berkov, S.; Font-Bardia, M.; Calvet, T.; Konrath, E.; Bueno, K.; Sachett, G.; Dutilh, J.H.; de Souza Borges, W.; Viladomat, F.; Henriques, A.T.; Nair, J.J.; Zuanazzi, J.A.S.; Batida, J. The Brazilian Amaryllidaceae as a source of acetylcholinesterase inhibitory alkaloids. *Phytochem. Rev.* **2015**. *15*, 147-160.

11. Molander, M.; Christensen, S.B.; Jäger, A.K.; Olsen, C.E.; Rønsted, N. 6-*O*-Methylkrigeine, a new Amaryllidaceae alkaloid from *Nerine huttoniae* Schönland. *Nat. Prod. Res*. **2012**, *26*, 56-60.

12. de Andrade, J.P.; Guo, Y.; Font-Bardia, M.; Calvet, T.; Dutilh, J.; Viladomat, F.; Codina, C.; Nair, J.J.; Zuanazzi, J.A.S.; Bastida, J. Crinine-type alkaloids from *Hippeastrum aulicum and H. calyptratum. Phytochemistry*, **2014**, *103*, 188-195.

13. Torres, J.C.; Pinto, A.C.; Garden, S.J. Application of a catalytic palladium biaryl synthesis reaction, via C-H functionalization, to the total synthesis of Amaryllidaceae alkaloids. *Tetrahedron* **2004**, *60*, 9889-9900.

14. Cedrón, J.C.; Gutiérrez, D.; Flores, N.; Ravelo, Á.G.; Estévez-Braun, A. Synthesis and antiplasmodial activity of lycorine derivatives. *Bioorgan. Med. Chem.* **2010**, *18*, 4694-4701.

15. Lamoral-Theys, D.; Andolfi, A.; van Goietsenoven, G.; Cimmino, A.; le Calvé, B.; Wauthoz, N.; Mégalizzi, V.; Gras, T.; Bruyère, C.; Dubois, J.; Mathieu, V.; Kornienko, A.; Kiss, R.; Evidente, A. Lycorine, the main phenanthridine Amaryllidaceae alkaloid, exhibits significant antitumor activity in cancer cells that display resistance to proapoptotic stimuli: an investigation of structure-activity relationship and mechanistic insight. *J. Med. Chem.* **2009**, *52*, 6244–6256.

16. Pettit, G.R.; Meng, Y.; Herald, D.L.; Knight, J.C.; Day, J.F. Antineoplastic agents. 553. The Texas grasshopper *Brachystola magna*. *J. Nat. Prod*. **2005**, *68*, 1256-125.

17. Masi, M.; van der Westhuyzen, A.E.; Tabanca, N.; Evidente, M.; Cimmino, A.; Green, I.R.; Bernier, U.R.; Becnel, J.J.; Bloomquist, J.R.; van Otterlo, W.A.; Evidente, A. Sarniensine, a mesembrine-type alkaloid isolated from *Nerine sarniensis*, an indigenous South African Amaryllidaceae with larvicidal and adulticidal activities against *Aedes aegypti. Fitoterapia* **2017**, *116*, 34-38.
